# Intransigent Vowel-Consonant Position in Korean Dysgraphia: Evidence of Spatial-Constructive Representation

**DOI:** 10.1155/2007/751407

**Published:** 2007-05-30

**Authors:** HyangHee Kim, Duk L. Na, Eun Sook Park

**Affiliations:** ^1^Graduate Program in Speech and Language PathologyYonsei UniversitySeoulKorea; ^2^Department of Rehabilitation MedicineYonsei University College of MedicineSeoulKorea; ^3^Department of NeurologySamsung Medical CenterSungkyunkwan University School of MedicineSeoulKorea

**Keywords:** Dysgraphia, writing, Korean, han-geul, English, consonant, vowel, spatial-constructional, internal orthographic representation

## Abstract

Dysgraphia due to a focal brain lesion can be characterized by substitution, transposition, deletion and/or addition errors of graphemes or strokes. However, those linguistic errors can be language-specific because the writing system of a given language may influence error patterns. We investigated a Korean stroke patient, a 57-year-old English teacher with dysgraphia both in Korean Han-geul (한글) and in English alphabet writings. The results of an experimental testing revealed transposition errors between a consonant and a vowel only in English but not in Korean writings. This austerity of vowel-consonant position may be attributed to a unique Korean writing system of a spatially well-formed syllabic configuration or block with consonant(s) and a vowel. In light of a neuropsychological model of writing, which depicts a multi-level spelling and writing process, we suggest a spatial-constructional component of internal orthographic representations in Korean writing. This Korean graphemic configuration feature may be resistant to a focal, left cerebral damage, and thus, we also discuss our results in terms of cerebral lateralization of the writing processes.

## 1. Introduction

Classical information processing models of writing and spelling depicts spelling and writing processes as consisting of multiple cognitive components [14]. Specifically, writing to dictation of familiar words requires the following processes. First, we analyze the acoustic input and compare the input signal with auditory input lexicon via auditory analysis system to recognize if the input is a familiar spoken word. Second, phonological input lexicon as the source of a specification of phonemic representations of the word is activated. Third, semantic system activates semantic features of the word. Fourth, abstract orthographic representations corresponding with the orthographic output lexicon, are retrieved from long-term memory. Here, the abstract orthographic representations are temporarily stored in a graphemic (or orthographic) output buffer, which refers to a working memory system. Finally, the graphemic output lexicon is converted into a specific letter string upon writing.

Although the nature of the internal orthographic representations remains obscure, certain hypotheses regarding some aspects of its structure have been accepted without much debate. For example, orthographic representations specify grapheme-level representa-

[1]This work has been presented at the 2006 American Speech-Language-Hearing Association (ASHA) Convention held in Miami, Florida, USA.

ISSN 0953-4180/07/$17.00 © 2007 – IOS Press and the authors. All rights reserved tions as a form of abstract letter identities and order among graphemes [3]. Therefore, patients with intact orthographic representations would identify if a given stimulus word is English or Korean. In addition, they would recognize if a stimulus word is written upside down. With graphemic buffer impairment, however, patients would show errors that took the form of substitutions, transpositions, deletions, and/or addition of grapheme(s) within a word stimulus [2,4]. Here, transposition errors are represented as an exchange of graphemes in their positions within a given stimulus.

While some writing processes might be universally common across languages, there may be certain writing features specific to a given language. It can be hypothesized that patients using different languages exhibit distinct graphemic error patterns upon writing due to dissimilar orthographic characteristics. In order to provide a framework for more detailed error analysis of writing in different languages, we would like to examine the writing systems of a few languages. For instance, Indo-European languages, such as English and Italian, utilize alphabetic scripts, which are characterized by a left-to-right sequence of letters. Further, the alphabets are written in a horizontal manner without any visually segmented syllabic structures. On the other hand, some of the Asian writing systems, such as Chinese characters, are logographic in nature [12, 17]. Each character is monosyllabic corresponding to a phoneme and is composed of a spatial configuration of multiple strokes (e.g., 馬). Meanwhile, Korean script /han-geul/[2] (한글) is unique in that it accommodates characteristics of both English script and Chinese characters. Here, we would like to introduce two features of /han-geul/. First, in contrast to Chinese writing systems, most Korean graphemes within a syllable correspond to Korean phonemes. For example, in the word 'san' (산, meaning 'mountain'), each grapheme, ㅅ, ㅏ, and ㄴ corresponds to phonemes, s, a, and n, respectively. This feature is very similar to English writing, although the phoneme-to-graphemic conversion rule is orthographically more consistent and less ambiguous in Korean. King Sejong invented the Korean writing system in the 15th century. A total of approximately 70 million South and North Koreans speak and write /han-geul/ on the Korean peninsula. As shown in Fig. 1, the Korean syllabic writing system has spatially well-formed syllabic blocks. Each block consists of an initial consonant, /cho-seong/ (초성), a medial vowel, /jung-seong/ (중성), and a final consonant, /jong-seong/ (종성). There are 24 letters, jamo (14 consonants and 10 vowels), in the Korean alphabet. Any Korean syllable can be created combining the consonants and vowels. The cardinal or radical components of a syllable are /cho-seong/ and /jung-seong/. In other words, /jong-seong/ can be optional to form a syllable. The locality of the medial vowel /jung-seong/ can be on the right of the first consonant /cho-seong/ or on the bottom of it. Graphemic shapes of /cho-seong/ would be altered depending on its location within a syllable. Second, unlike English script where graphemes run only in left-to-right orientation, the Korean script has a spatial combination of multiple graphemes of left-to-right, top-to-bottom, and/or two mixed orientations within a syllable. This feature is similar to the organization of a Chinese character.

It is speculated that this Korean orthographical uniqueness may differentially influence error patterns in Korean writing. However, in comparison with dysgraphia in other languages, Korean dysgraphia has received relatively little attention from worldly scholars. To this date, there have been a few studies on Korean dysgraphia [9,10]. In this paper, we propose to address a unique, spatial-constructional component of orthographic representation in Korean writing. In order to provide evidence, we compared Korean error patterns with those in English writings of a Korean patient with a focal left hemisphere lesion.

## 2. Methods

### 2.1. Participant

YK was a 57-year-old right-handed man with a college education. For the last 27 years, he has worked as an English teacher in local senior-high schools. He suffered from a left middle cerebral artery (MCA) embolic infarction. As seen in Fig. 2, the results of the brain magnetic resonance imaging (B-MRI) revealed lesions in the left pre-motor area (Brodmann area 6), insular cortex and superior parietal lobule. YK had been referred for a comprehensive speech-language evaluation by a neurologist.

[2]All broad phonetic transcriptions are written in '/ /' and are provided according to a romanization converting program developed by '21st Century Sejong Project' funded by Ministry of Culture and Tourism of Korea. The authors took the liberty of inserting ' – ' between syllables for the sake of demonstrating visual segmentation.

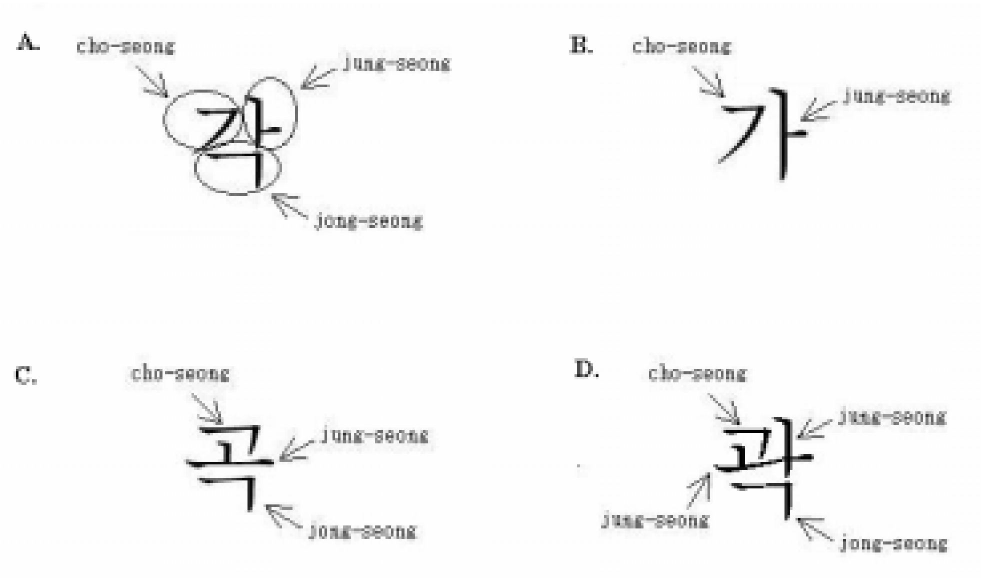

Fig. 1. The writing system of a Korean syllable. **A**: initial (ㄱ), medial (ㅏ), and final (ㄱ), graphemes are named as cho-seong, jung-seong and jong-seong, respectively. jung-seong (ㅏ) is written right of cho-seong (ㄱ); **B**: Notice that this syllable does not contain a jong-seong, but still remains as a syllable, demonstrating jong-seong can be an optional element of a syllable; **C**: Notice that the shape of cho-seong is altered (ㄱ → ㄱ) on this syllable as a consequence of the locus change of jung-seong from the right (ㅏ) to the bottom (ㅜ) of cho-seong; and **D**: This syllabic block has two jung-seong graphemes written wrapping around cho-seong.

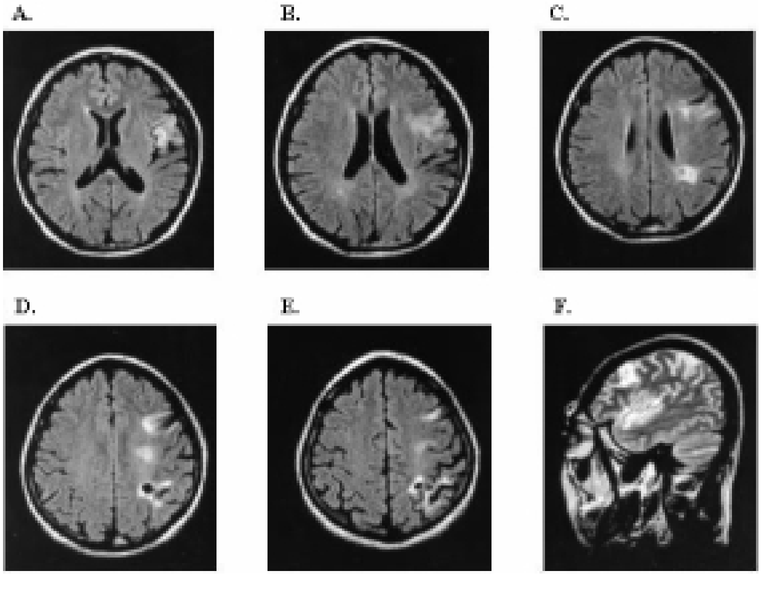

Fig. 2. The Axial FLAIR (**A** $\sim$ **E**) and sagittal T2 weighted (**F**) Brain MRI demonstrating high signal abnormalities involving the left premotor area (Brodmann area 6), insular cortex and superior parietal lobule.

### 2.2. *Language testing*

A formal language evaluation was performed 28 days post-stroke. Our initial language evaluation of YK with the Korean version of the Western Aphasia Battery (WAB) [11] revealed spontaneous speech scores of 18 out of 20 total, comprised of scores of 9 each in the ‘content’ and ‘fluency’ subcategory ratings. YK

performed 8.25 out of 10 in the auditory comprehension subtest. Repetition (9.1 out of 10) and naming (8.7 out of 10) scores were within the mild impairment range. His overall aphasia quotient was 88.3. His reading score was 90 out of 100 in the reading subtest of the WAB. The writing subtest score was 71 out of 100. Copying of written stimuli was intact. In the writing subtest, YK wrote with his dominant right hand. Written grapheme formation was somewhat weak but intact, revealing no apractic agraphia [1].

### 2.3. Experimental testing

Experimental testing took place on the following day of the language evaluation. In the task of writing 19 phonologically acceptable pseudowords (non-words), 19 real words and 19 irregular words with graphemic clusters, YK did not show any more difficulties in writing the pseudowords than writing real and irregular words. YK wrote two word-lists from dictation, one with Korean and the other with English words. Each word-list contained 110 two- or three- syllables words. Only 110 pure Korean words were analyzed for this study, although additional responses to Chinese-derivative words were collected because the two types of stimuli in writing were found to be different in nature [10]. Each syllable of 70 two-syllable words was composed of Consonant-Vowel (hereafter, CV), CVC, or CVCC. Word examples of CV + CVC structure was 'begin' in English, and /mu-deom/ (무덤) in Korean. For CVC+CVC structure, we included an English word such as 'filter' and Korean word such as /maep-ssal/ (맵쌀). The three-syllabic word-list contained 40 words with CVC + CVC + CVC (e.g., hamburger, /gang-nang-kong/ (강낭콩)), CVC + CV + CVC (e.g., computer, /bin-dae-tteok/ (빈대떡)), CV + CVC + CVC (e.g., reporter, /bi-bim-bap/ (비빔밥)) or CV + ACV + CV (e.g., banana, /du-deo-ji/ (두더지)) compositions. English-unique syllabic compositions such as CCV (e.g., 'sha' as in 'shadow') were excluded, because in Korean, consonant clusters with two diverse consonants (e.g., ㄳ) are not placed in the initial syllable position, /cho-seong/ ( 초성 ). YK was asked to rehearse each dictated word prior to writing, in order to verify intact auditory input of each stimulus. No prompts were given to YK in the processes of writing. The testing procedure was videotaped in order to verify his written processes and responses, if necessary.

## 3. Results

The written responses were analyzed by the frequency of errors on each word list. As seen in Table 1, with both Korean and English word stimuli, the error rate increased as the length of stimuli increased. With two-syllable stimuli, Korean and English error rates were 54.2% and 52.9%, respectively, and the error rates for three-syllable stimuli were 95.9% and 75.0%, respectively. Further, a Pearson chi-square test revealed that significantly more frequent errors in Korean compared to English stimuli were observed in three-syllable stimuli ($\chi^2 = 4.627$; df $= 1$; $p = 0.031$), but not in two-syllable stimuli.

As shown in Table 2, four types of errors were analyzed, namely, substitution, transposition, deletion and addition errors. If a word stimulus contained multiple erroneous syllables, all syllables with errors were counted, resulting in 150 erroneous syllables in Korean and 178 in English words. The four error types were not evenly distributed. Substitution errors were the most common in both languages: 71.3% and 41.6% in Korean and English, respectively. Errors of transposition between graphemes were the second most common in both languages: 12.0% and 30.3% in Korean and English, respectively. However, vowel-consonant transposition errors only occurred with English stimuli and not with Korean stimuli. The least observed errors for both languages were addition errors. Addition error rates were 6.7% and 9.6% for Korean and English stimuli, respectively, whereas deletion errors rates were 10.0% for Korean stimuli, and 18.5% for English stimuli. The results of the Pearson chi-square tests revealed significantly more substitution errors with Korean writings than with English writings ($\chi^2 = 15.976$; df $=$ 1; $p = 0.000$). In contrast, we observed significantly more transposition errors ($\chi^2 = 4.627$; df $= 1$; $p =$ 0.000) and deletion errors ($\chi^2 = 4.752$; df $= 1$; $p =$ 0.029) with English writings. However, there was no significant difference in number of addition errors between the two languages. Table 3 shows examples of four error types in Korean and English writings.

## 4. Discussion

Given the following three aspects of the results, we demonstrated that YK manifested a graphemic buffer deficit with errors in both Korean and English stimuli. First, in a pre-test, YK displayed very similar error rates across different stimulus types, pseudowords, re-

Table 1
Error rates (%) (Number of stimuli with errors/Total number of stimuli) in Korean and English writings depending on stimuli length

| Language Stimuli length | Korean | English | Total |
|---|---|---|---|
| Two-syllable | 54.3% (38/70) | 52.9% (37/70) | 54% (75/140) |
| Three-syllable* | 95.0% (38/40) | 75.0% (30/40) | 85% (68/80) |
| Total | 69.1% (76/110) | 60.9% (67/110) | |

$^{*}p < 0.05$.

Table 2
Error rates (%) according to types of errors (Number of errors/Total number of errors) in Korean and English letter writings[a]

| Language Error types | Korean | English |
|---|---|---|
| Substitution** | 71.3% (107/150) | 41.6% (74/178) |
| Transposition[b],** | 12.0% (18/150) | 30.3% (54/178) |
| Deletion* | 10.0% (15/150) | 18.5% (33/178) |
| Addition | 6.7% (10/150) | 9.6% (17/178) |
| Total | 100% (150/150) | 100% (178/178) |

[a] If a word stimulus contains more than two letter errors, all errors were counted.
[b] If one graphemic transposition occurs, it was counted as two errors.
$^{*}p < 0.05$. $^{**}p < 0.01$.

al words and irregular words. Specifically, he did not show any greater difficulties in writing pseudowords as opposed to real and irregular words. Second, as stimulus length increased from two to three syllables, the error rates increased in English as well as Korean writings. This finding may be indicative that the graphemic buffer serves as a working memory [2]. In this study, we did not administer additional tasks (e.g., high vs. low frequency words; noun vs. verb) to further testify the graphemic buffer impairment in YK because it was not tenable to match the linguistic features (e.g., frequency of occurrence) between the two languages. It is uncertain why YK demonstrated more frequent errors in three-syllablic Korean words compared to three-syllablic English words. However, the fact that we employed some three-syllablic Korean stimuli requiring irregular phoneme-to-grapheme conversion due to glottalization (e.g., /g/ ㄱ can be glottalized as /gg/ ㄲ as in word /jang-nan-ggam/ 장난감– meaning 'toy') might, in part, explain the phenomenon. Third, we could observe four types of writing errors, primarily with substitution and transposition errors, that Caramazza and his colleagues claim is evidence of graphemic buffer impairment [3,8]. After the selection of the correct phonological code, the sequence of phonemes was misguided. These two errors were common with both Korean as well as English writings. It has been reported that the most frequent error type produced by an Italian patient was letter substitutions [3].

From the results of the error type analysis, a unique feature of transposition errors in Korean writing was observed. Specifically, there were less frequently occurring transposition errors in Korean writing relative to English writing. YK never exchanged an initial or final consonant with a cardinal medial vowel within or across syllabic structure(s). Instead, transposition errors occurred only between consonants or between vowels across syllables. For example, as seen in Table 3, the Korean word /me-a-ri/ (메아리) was written as /me-i-ra/ (메이라), the vowel 'a' being exchanged with the vowel 'i' across syllabic blocks, not with a consonant. On the other hand, in English writing, YK often showed transposition errors between a consonant and a vowel. For example, the word 'computer' was erroneously written as 'com*up*tom' with 'p' and 'u' being interchanged. When we additionally presented the subject several syllabic compositions with two subsequent vowels (e.g., CVVC + CVC: 'counter'), which are unique to English, the word 'counter' was written as 'co*nu*ter' and 'accident' as 'acc*di*tent', resulting in vowel-consonant transposition errors.

These errors in the English writing may occur because alphabet scripts, such as English and Italian, are horizontally written letter strings without spatial-constructional, syllabic boundaries. In turn, the locality of vowels and consonants within a word may not need to be rigid. In fact, an Italian patient reported in a study by Caramazza and Miceli made 20% of vowel-consonant transposition errors [3]. The investigators asserted that the exchange errors certainly violated the CV structure of the target response. This observation was further discussed by Cubelli, who also reported 10% of transposition errors, errors involving exchanges of vowels for consonants and consonants for vowels in his Italian dysgraphic patient [5]. Although the investigators in both studies claimed vowel-consonant status as a multi-dimensional structure in graphemic representations, the vowel-consonant status in languages with alphabet strings seems to not be as rigid as that of Korean writing.

It was unique that there were not vowel-consonant transposition errors in YK's Korean writings. A likely explanation is that the Korean vowel 'jung-seong' as a cardinal element in a syllabic structure is maintained

Table 3
Examples of error types in Korean and English writings[a]

| Language | Korean | | English | |
|---|---|---|---|---|
| Error types | Response | Target | Response | Target |
| Substitution |  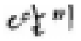 /ttal-ggi/ | 딸기 /ttal-gi/ |  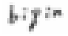 | b*e*gin |
| Transposition |  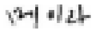 /me-*i*-*ra*/ | 메아리 /me-a-ri/ |  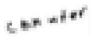 | co*un*ter |
| Deletion |  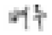 /byeo-ru/ | 벼룩 /byeo-ru*k*/ |  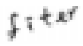 | fi*l*ter |
| Addition |  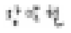 /jang-ma*l*-cheol/ | 장마철 /jang-ma-cheol/ |  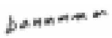 | banana |

[a] Erroneous letters are written as *italic* on either responses or targets.

in a linguistic as well as spatial-constructional position. First, in terms of a linguistic-phonological point of view, it has been reported that, in Korean, word-processing is accomplished via inner speech centering around a vowel [16]. This feature may be observed in any language with syllabic, phonological segmentation. However, Korean syllabic structure with a cardinal vowel and a mandatory initial consonant may require firmer phonological encoding upon production. Second, from the spatial-constructional point of view, syllabic blocks of Korean syllables may play an important role in retaining the position of vowel and consonant. Specifically, the locus of 'jung-seong' within a syllable would be ingrained as a visual-spatial image while learning the Korean writing system. Thus, upon a writing task, the mental imagery in the orthographic representations would be encoded to produce a writing construct, such as with a task of free drawing [6]. In addition to phonological segmentation of a word, this spatial-constructional segmentation may enhance writing integrity. In contrast, the orthographic construction words without visually segmented boundaries of syllables in English writing would be inclined towards vowel-consonant transposition errors. Although some researchers reported a rigid vowel-consonant status in some English [7] and Italian-speaking case studies, their subjects were not without any vowel-consonant transposition errors. This uniqueness of linguistic as well as a spatial-constructional component in Korean syllabic writing may support a notion of orthographic representations as multi-dimensional structures, as claimed by Caramazza and Miceli [3]. It may be proposed that the abstract orthographic representations of the Korean writing system accommodates spatial-constructional configuration of graphemes.

This spatial-constructional component may be a function of the right hemisphere [18]. Therefore, it is thought that the representation is preserved in patients with damage in the left cerebral hemisphere. YK only had lesions in the left hemisphere, including the frontal and parietal lobes, which are related to the working memory system of the frontal-parietal circuit [13,15]. Written spelling may be a function of the left hemisphere, whereas the spatial-constructive component of writing may be a function of the right hemisphere.

## 5. Conclusion

The significance of this study is the observation of the austerity of the vowel-consonant locus in Korean writing. Therefore, we propose a spatial-constructional, configurative component of abstract internal representations that may be unique to the Korean writing system.

## Acknowledgement

The authors are grateful to Dr. Argye Hillis at Johns Hopkins University School of Medicine and the anonymous reviewer for the excellent suggestions that considerably improved the manuscript.
